# Biomaterials delivery strategies to repair degenerated intervertebral discs by regulating the inflammatory microenvironment

**DOI:** 10.3389/fimmu.2023.1051606

**Published:** 2023-01-23

**Authors:** Yuanliang Xia, Hengyi Wang, Ruohan Yang, Yulin Hou, Yuehong Li, Jianshu Zhu, Changfeng Fu

**Affiliations:** ^1^ Department of Spine Surgery, The First Hospital of Jilin University, Changchun, China; ^2^ Cancer Center, The First Hospital of Jilin University, Changchun, China; ^3^ Department of Cardiology, Guangyuan Central Hospital, Guangyuan, China

**Keywords:** intervertebral disc degeneration, lower back pain, nucleus pulposus, annulus fibrosus, biomaterials

## Abstract

Intervertebral disc degeneration (IVDD) is one of the leading causes of lower back pain. Although IVDD cannot directly cause death, it can cause pain, psychological burdens, and economic burdens to patients. Current conservative treatments for IVDD can relieve pain but cannot reverse the disease. Patients who cannot tolerate pain usually resort to a strategy of surgical resection of the degenerated disc. However, the surgical removal of IVDD can affect the stability of adjacent discs. Furthermore, the probability of the reherniation of the intervertebral disc (IVD) after surgery is as high as 21.2%. Strategies based on tissue engineering to deliver stem cells for the regeneration of nucleus purposes (NP) and annulus fibrosus (AF) have been extensively studied. The developed biomaterials not only locally withstand the pressure of the IVD but also lay the foundation for the survival of stem cells. However, the structure of IVDs does not provide sufficient nutrients for delivered stem cells. The role of immune mechanisms in IVDD has recently become clear. In IVDD, the IVD that was originally in immune privilege prevents the attack of immune cells (mainly effector T cells and macrophages) and aggravates the disease. Immune regulatory and inflammatory factors released by effector T cells, macrophages, and the IVD further aggravate IVDD. Reversing IVDD by regulating the inflammatory microenvironment is a potential approach for the treatment of the disease. However, the biological factors modulating the inflammatory microenvironment easily degrade *in vivo*. It makes it possible for different biomaterials to modulate the inflammatory microenvironment to repair IVDD. In this review, we have discussed the structures of IVDs and the immune mechanisms underlying IVDD. We have described the immune mechanisms elicited by different biological factors, including tumor necrosis factors, interleukins, transforming growth factors, hypoxia-inducible factors, and reactive oxygen species in IVDs. Finally, we have discussed the biomaterials used to modulate the inflammatory microenvironment to repair IVDD and their development.

## 1 Introduction

Intervertebral disc degeneration (IVDD) is a common clinical form of spinal degeneration. Inflammation, oxidative stress, and mechanical stimulation may lead to an imbalance of the anabolic and catabolic processes in the extracellular matrix (ECM) of intervertebral discs (IVDs) and loss of nucleus pulposus (NP), leading to IVD dysfunction and structural damage (1, 2). IVDD leads to the partial or complete rupture of annulus fibrosus (AF). The NP protrudes backward from the ruptures, irritating or compressing the nerve root and causing low back and leg pain (3). IVDD is one of the leading causes of chronic low back pain (LBP) in patients (4). Clinically, approximately 40% of LBP cases are due to discogenic causes (5). Although IVDD does not have a fatal impact on patients in the short term, it is a significant cause of disability and socioeconomic stress (6). In the U.S. alone, for example, indirect and direct costs of LBP costs over $100 billion (7).

IVDs are the fundamental motor units of the spine. They have high compressive and tensile strengths, which can maintain the axial pressure of the spine and ensure the axial flexibility of the body (8). IVDD is an abnormal, cell-mediated response to progressive structural damage (9). The decreased height of the degenerative IVDs changes the stress-bearing spine segment and accelerates the degeneration of other adjacent segments. The late stage of IVDD causes the chronic instability of spinal segments, which seriously affects the quality of life of patients (10). The prevalence of IVDD is rising owing to risk factors such as aging, obesity, chronic stress, and smoking (7). Currently, the leading conservative treatment methods for IVDD are anti-inflammatory analgesia and physical therapy (11). Conservative treatments can only delay degeneration, slow down the rate of IVD degradation, and temporarily relieve pain, but cannot reverse IVDD (12). Patients with advanced IVDD can undergo surgical decompression and disc replacement (13). However, surgical resections of IVDs can still lead to re-protrusion. In a 2015 meta-analysis, the reherniation rate after lumbar disc herniation was found to be as high as 21.2% (14). Surgical treatments aim for symptomatic relief but increase the pressure and risk of injury to discs adjacent to the affected IVD (15).

Current regenerative IVD technologies based on tissue engineering have achieved encouraging results (16). For example, biomaterials for the repair of NP and AF have regenerated IVDs in animals by carrying mesenchymal stem cells (MSCs) like bone marrow-derived mesenchymal stem cells (BMSCs), adipose-derived mesenchymal stem cells (ADSCs) and induced pluripotent stem cells (iPSCs) (17–23). Cartilage endplate-derived stem cells (CESCs), annulus fibrosus-derived stem cells (AFSCs), and nucleus pulposus-derived stem cells (NPSCs) can also replace the IVD (21, 24–26). IVDD can also secrete Stromal cell-derived factor-1α (SDF-1α) to induce the chemotaxis of NPSCs to the NPs center for *in situ* regeneration (27). IVD regeneration is mainly achieved by inducing the differentiation of MSCs into NPCs or AFCs (28–30). Although MSCs have achieved good results in bone and cartilage regeneration, similar effects cannot currently be exerted in IVDD, even if the MSCs were delivered to IVDs, due to the lack of vascular systems in the discs (31). This is because MSCs cannot absorb sufficient nutrients during IVDD. The harsh environment of IVDD is unsuitable for NPSCs and MSCs to proliferate and repair NPs. Although current cell transplantation technologies can regenerate IVDs, cell transplantation strategies remain insufficient, and the regeneration of IVDs cannot be completed in a time-efficient manner (32).

**Figure 1:**
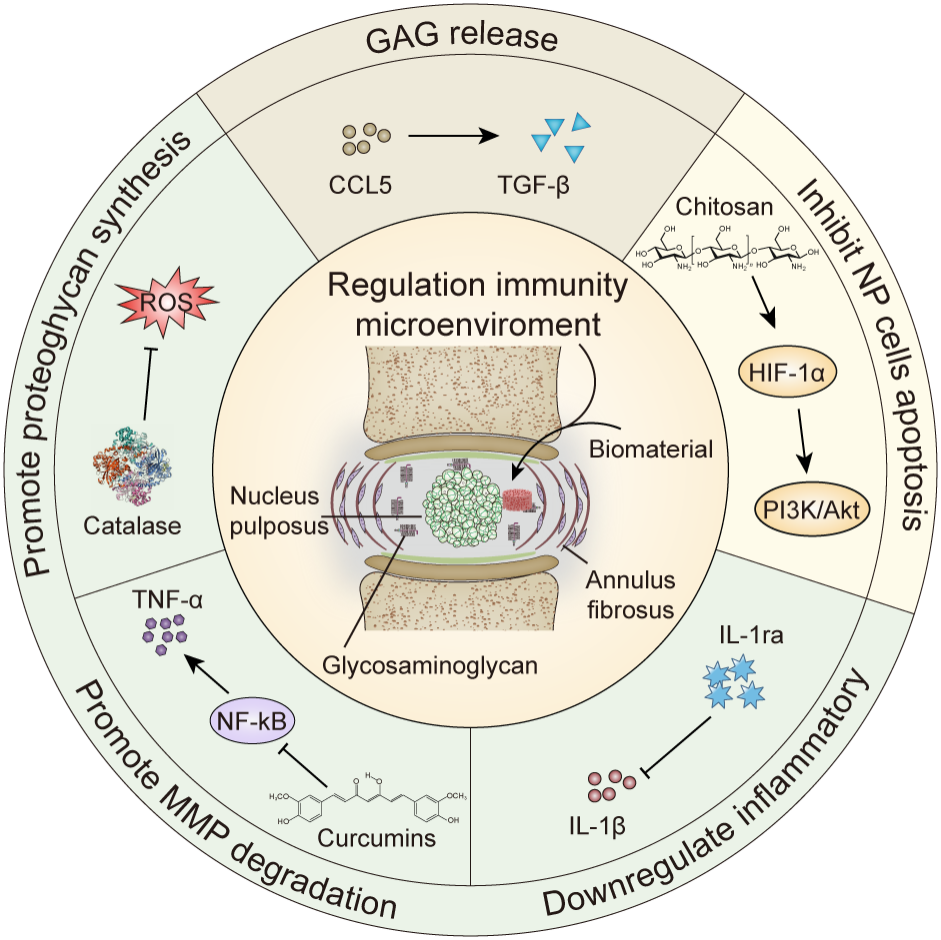
Biomaterials regulate cytokine repair of intervertebral disc degeneration. GAG, glycosaminoglycan; MMP, matrix metalloproteinase; HIF-1α, hypoxia inducible factor-1α; IL-1ra, Interleukin -1 receptor antagonist; TNF, tumor necrosis factor; ROS, reactive oxygen species.

Studies have shown that when IVDs degenerate, NP that initially maintained immune tolerance become targets of immune system attacks (33, 34). T cells, macrophages, and inflammatory factors like interleukin (IL)-1β, tumor necrosis factor (TNF)-α) are recruited around NP. The inflammatory response caused by IL-1β, TNF-α, and reactive oxygen species (ROS) further amplifies the inflammatory response locally and accelerates IVDD (35). Impaired AF and prominent NP cause IVD to lose its physical barrier, and IVD is fully exposed to the immune system. While removing cellular debris and foreign bodies, T cells and macrophages destroy normal AF and NP, further exacerbating damage to IVD. Macrophages are divided into two types according to their role: M1 type and M2 type. M1 macrophages are pro-inflammatory cells, and M2 macrophages are anti-inflammatory cells. While M1 macrophages have the effect of phagocytosing necrotic matter in the early stages of inflammation, excessive inflammation can exacerbate the progression of IVDD. M2 macrophages are mainly manifested as promoting tissue repair (33). Therefore, the inhibition of IVDD by regulating the inflammatory microenvironment is feasible. However, biological factors that regulate immunity (such as growth and inflammatory factors and chemokines) are easily degraded *in vivo* and cannot meet the long-term requirements for IVDD repair (5, 36). Furthermore, IVDs do not have a vascular system and cannot transport biological factors through systemic administration. A feasible way to repair IVDD is to inject biological factors into IVDs by designing biomaterials. Biomaterials based on chitosan and alginate have good biocompatibility and can not only treat IVDD by regulating the inflammatory microenvironment but also by carrying immune regulators (34, 37). In this review, we have discussed the structures of IVDs, the immune mechanisms of IVDD, and the effects of inflammatory immune factors such as IL-1β and TNF-α on IVDD. Finally, we have discussed the various biomaterials that regulate IVDD repair in the inflammatory microenvironment (**Figure 1**; **Table 1**) and their development.

**Table 1:** Biomaterials regulate the inflammatory microenvironment to repair degenerated intervertebral discs.

Regulator	Biomaterial	Production Method		Therapeutic components	Target	Result	Reference
TGF	Fibrin hydrogels	Fibrinogen		CCL5	AF	Fibrin hydrogels release CCL5 for the chemotaxis homing effect of AF cells, but do not promote AF cell repair in sheep.	(38)
TGF	pullulan microbeads	Fitc-pullulan was dissolved in 20 mL of distilled water under magnetic stirring and lyophilized after the addition of a cross-linking agent. Pullulan microbeads (PMBs) and 500 u of LCCL5 and TGF-β1 were magnetically stirred for 24 h.		CCL-5 TGF-β1	ECM	PMBs were loaded with CCL5/TGF-β1/GDF-5 continuously and sustainably released growth factors and maintained their biological activities *in vitro.* Increased distance that ASCs can migrate to NP.	(39)
TGF	Collagen-Polyurethane scaffold	Polyurethane (PU) was dissolved in N,N-dimethylformamide solution, and the PU film was formed after evaporating the solvent. Type I collagen fibers were used as cell carriers to encapsulate AF cells and TGF-β1 on PU membranes to make collagen-PU scaffolds.		AF cells TGF-β1	AF ECM	The TGF-β1 treatment of collagen hydrogels further promotes cell proliferation and matrix production in AF cells *in vitro.*	(40)
	chitosan (CH)- based scaffold	Mixing chitosan with porcine gelatin at a ratio of 3:2 can further increase the biological response of encapsulated cells by adding gelatin and Link N (a peptide present in IVDs).		Link	ECM	LN increased GAG production in degenerative media to the same level as that of TGF-β. The addition of 1% gelatin to the CH hydrogel further increased GAG production *in vitro.*	(41)
	TGF-β3@PDA NPs	PDA NP were prepared through chemical oxidative polymerization using dopamine (DA) and tris-(hydroxymethyl)-aminomethane (Tris). TGF-β3 was loaded onto the surface of PDA nanoparticles in a covalent binding manner.		TGF-β3	AF NP	The released GFs could induce the differentiation of BSMCs into myeloid and annulus-like cells and maintain high activity. Finally, *in vivo* experiments confirmed that the reconstituted IVD scaffolds exhibited region-specific stromal phenotypes with histological and immunological features.	(42)
	Genipin Fibrin Gel	Genipin and fibrinogen in a humidified incubator for 3–4 h for polymerization and cross-linking.		TGF-β3	ECM	Fibrin increases integrin binding sites and prevents partially encapsulated cells from undergoing apoptosis, allowing encapsulated cells to increase ECM synthesis *in vitro.*	(43)
	gelatin-hyaluronic acid methacrylate (GelHA) hydrogel	Methacrylic anhydride (MA) was added to the gelatin solution and allowed to react for 1 h. The resulting solution was dialyzed for 3 days and lyophilized. Photoinitiators and integrins were added, and photocrosslinking was performed under UV light irradiation for 2 min.		integrin	ECM	The combination of photocrosslinked GelHA hydrogel and ASC can enable ASC to undergo NP-like differentiation and enhance the efficacy of ASC for IVD repair by activating the integrin αvβ6-TGF-β1 pathway *in vitro.*	(44)
IL-α	HA-pNIPAM	Gelatin was combined with EGCG, mixed with water, and stirred at 40°C for 4–6 h. Poly-N-isopropylacrylamide was directly grafted onto HA. Gelatin was directly applied to (HA-pNIPAM).		EGCG	ECM	EGCG microparticles combined with a suitable carrier can modulate the activity/release of EGCG in the IVD *in vitro.*	(45)
IL	HMw-HA Gel	HMw sodium hyaluronate was dissolved in 1 mL of distilled water and mixed with PGE-amine to induce cross-linking to obtain spherical hydrogels.		–	ECM	The hydrogels inhibited the expression of the inflammatory receptors IL-1R1 and MyD88, downregulated NGF and BDNF gene expression, and upregulated CD44 receptor expression *in vitro.*	(46)
IL	PLGA Microspheres	After mixing IL-1ra with 1 mL of 75:25 PLGA and sonicating the mixture, 1% polyvinyl alcohol (PVA) magnetic stir bar was added and mixed for 3 h.		IL-1ra	NP	IL-1ra delivered from PLGA microspheres effectively attenuated IL-1β-mediated inflammatory changes in engineered NP constructs *in vitro.*	(47)
IL	Chitosan/Poly-γ-glutamic acid nanoparticles	Chitosan/Poly-γ-glutamic acid was mixed at a molar ratio of 1:1.5 to prepare nanoparticles by the co-coagulation method. Diclofenac (Df) was added to the nanoparticles and stirred at a constant speed.		Df	ECM	The intradiscal injection of Ch/Df/γ-PGA NPs reduced pro-inflammatory mediators, downregulated MMP 1 and 3 expression, and upregulated Col II and Agg production in a pro-inflammatory/degenerative IVD organ culture model *in vitro.*	(48)
TNF	ELP-curcumin conjugates	Elastin-like polypeptide (ELP) is a thermoresponsive biopolymer composed of Val-Pro-Gly Xaa-Gly pentapeptide repeating units. Curcumin is chemically modified and coupled with ELP.		Curcumin	ECM	The ELP-curcumin conjugate rapidly forms a depot after physiological administration and slowly releases bioactive curcumin in the perineural space to treat neuroinflammation *in vitro.*	(49)
	pNIPAAM MgFe-LDH Gel	N-isopropylacrylamide forms a pNIPAAM polymer *via* free radical polymerization. Polymers with MgFe layered double hydroxide (LDH) nanoparticles and CXB were dissolved in water.		CXB	ECM	The controlled release of CXB from this hydrogel resulted in the inhibition of PGE 2 in a mice model of spontaneous IVD degeneration.	(50)
HIF	Small leucine-rich proteoglycans	–		–	ECM	Biglycan can bind and (TGF-β) to activate the MAPK pathway to enhance HIF-1α translation *in vitro.*	(51)
	Chitosan-alginate gel scaffold	Chitosan was dissolved in acetic acid, and after filtering the solution, the pH was adjusted to 8.5 with 0.1 mol/L NaOH. The sterile alginate solution was then mixed with the chitosan solution at a ratio of 1:1.		ADSC	NP ECM	ADSCs grew well in the C/A gel scaffolds, differentiated into NP-like cells under certain induction conditions, produced the sameECM as NP cells, and were promoted under hypoxia *in vitro.*	(52)
	Nanofibrous spongy microspheres	Development of poly (L-lactic acid) grafted poly(hydroxyethyl methacrylate) (PLLA-g-PHEMA) nanoparticles using the phase separation method. miR-199a was encapsulated in nanoparticles using the double emulsion technique.		miR-199a	NP	Sustained release of *in situ* anti-miR-199a inhibits miR-199a, which in turn enhances HIF-1α and Sox-9 activity, thereby inhibiting calcification and promoting NP regeneration in mice.	(53)
	Polymer capsule	Calcium carbonate was used as a sacrificial template to fabricate catalase-loaded polymer capsules functionalized with an outer layer of tannic acid (TA) by a layer-by-layer approach.		Catalase	ECM	ROS-responsive polymer capsules reduce the potential for oxidative stress and downregulate MMP expression in the ECM.	(54)
ROS	Rapamycin hydrogel	The ROS-labile linker was synthesized *via* the quaternization reaction of tetramethylpropane-1,3-diamine with an excess of 4-(bromomethyl) phenylboronic acid. Cross-linking of the ROS-labile linker with poly(vinyl alcohol) (PVA) to form ROS-scavenging hydrogels for loading rapamycin.		Rapamycin	ECM	ROS-responsive hydrogel scaffolds and rapamycin can reduce ROS levels and promote macrophage polarization to M2 type *in vitro.*	(55)
	Alginate scaffold	The sodium alginate solution was diluted with sterile saline, then the Perfluorotributylamine emulsion was added and sonicated.		–	NP ECM	Perfluorotributylamine -enriched alginate scaffolds promote NP cell survival and proliferation *in vitro.* Furthermore, Perfluorotributylamine can modulate ECM expression to generate disc-like tissue grafts in mice.	(56)

GAG, glycosaminoglycan; MMP, matrix metalloproteinase; HIF-1α, hypoxia inducible factor-1α; IL-1ra, Interleukin -1 receptor antagonist; TNF, tumor necrosis factor; ROS, reactive oxygen species; ECM, extracellular matrix; CCL-5, chemokine (C–C motif) ligand; ELP, elastin-like polypeptide; CXB, celecoxib; NGF, nerve growth factors.

## 2 Intervertebral discs and their mechanisms in inflammatory-immunity

IVDs are the connecting parts and are the most fundamental functional units of the spine. IVDs consist of three tissue types: the central gelatinous NP, surrounding AF tissue, and upper and lower cartilage endplates (CEPs). The IVD cartilaginous tissue in the spine acts as a shock absorber, assists in the movement of vertebral bones, and holds the vertebrae together to coordinate the direction of the spine (57). The IVD has been identified as an immune privilege organ (34). As the IVD aging process or traumatic injury occurs, immune and inflammatory cells infiltrate the IVDs. These cells and cytokines produce pro-inflammatory substances while clearing necrotic cells, leading to disturbances in the inflammatory microenvironment and aggravating IVDD (58). Therefore, understanding the connection between the structure of IVDs and the immune system is crucial in elucidating the mechanisms of IVDD.

### 2.1 Nucleus pulposus

NP consists of water, type II collagen, chondrocyte-like cells, and proteoglycans. These structures make NP elastic to resist pressure on the spine and transmit the pressure to AF and surrounding CEPs (59). The absence of nerve cells or blood vessels within NP allows NP to become an immune-privileged organ (33). Recently, an increasing number of studies have shown that maintaining immune privilege requires using various molecular biology techniques. For example, NP can express the Fas ligand (FasL) to induce the apoptosis of T cells and macrophages to maintain their immune tolerance (60). In addition, FasL expressed by NP can also lead to the apoptosis of vascular endothelial cells. AF and CEPs tightly surround NP to isolate NP from the host immune system (34). These main mechanisms make normal NP immune to attacks by the immune system and contribute significantly to maintaining IVD stability.

As IVDD progresses, AF ruptures, and NP protrudes, so the barrier between the IVD and the immune system is broken. The damage exposes the NP that was originally immune-privileged to the immune system so that they become targets for immune system attacks (33). Nerve root compression and the autoimmune response to NP are the critical causes of radicular pain in the late stage of IVDD (58). A large number of macrophages, T cells, B cells, and natural killer (NK) cells and cytokines (such as fibroblast growth factors, IL family members, and TNFs) are recruited around nerve roots, causing nerve root immune stress pain (13, 34, 61). The infiltration of inflammatory cells removes necrotic cells. At the same time, excessive inflammatory cells also remove non-necrotic cells, which aggravates the destruction of IVD by the immune system (62). Studies have shown that macrophages are the inflammatory cells that infiltrate IVDs and are associated with IVDD progression (63). This is due to the upregulation and activation of p38 in IVDD. The activation of p38 in NP induces macrophage polarization and the expression of the catabolic enzyme matrix metalloproteinase-13 (MMP-13). Overexpressed MMPs destroy the ECM, thereby aggravating the degenerative process (63). The difference in macrophage phenotypes between degenerated and normal IVDs may be a novel target for therapies.

### 2.2 Annulus fibrosus

AF is highly fibrotic tissue that surrounds the exterior of the NP. AF mainly contains type I collagen produced by fibroblasts and contain type II collagen produced by fibrochondrocytes (64). This unique structure provides AF with good mechanical properties. AF tightly protect NP in the inner layer and provides a foundation for maintaining the structure of IVD (34). There are various stem cells present around AF. These stem cells contribute to maintaining the turnover of AF (65). With increasing age, the numbers of stem cells gradually decrease and become insufficient to repair damaged AF promptly, which is the main reason for the impaired healing of AF (66). After AF is damaged, T cells and macrophages are activated to clear the damaged tissue and debris. Intercellular adhesion molecule 1 (ICAM1) is an inducible surface glycoprotein that induces the adhesion, migration, and invasion of lymphocytes to sites of degeneration during immune responses (67). Studies have shown that chemokine ligand CCL-2 can differentiate monocytes into macrophages and that macrophage infiltration is a significant cause of IVDD-induced radicular pain (68). Although T cells and macrophages are recruited to clear damaged AF, T cells and macrophages also aggravate AF damage (66). This balance is difficult to grasp, but modulating it to tilt it in the desired direction may be a new target for IVDD immunotherapy.

### 2.3 Cartilage endplates

CEPs are thin layers of hyaline cartilage that separate IVDs from the upper and lower vertebral bodies. IVDs, composed of CEPs, NP, and AF, protect the bones of the vertebral body from mechanical damage and maintain the normal physiological movement of the spine. Unlike NP and AF, CEPs have a vascular system. The nutrient metabolism of IVDs is mainly dependent on the vascular system within CEPs (69). CEPs are composed of hyaluronic acid (HA), type II collagen, and PECM secreted by chondrocytes (70). The ECM is rich in stem cells, which are advantageous not only in osteogenesis and chondrogenesis (71) but also in replacing AF and NP. Studies have shown that HIF-1α is highly expressed in NP owing to hypoxia (72). HIF-1α induces the differentiation of cartilage endplate stem cells (ECSCs) into NP (73, 74). CEP-derived exosomes (N-Exos) reduce apoptosis of CEP by activating the phosphoinositide 3 kinase/protein kinase B (PI3K/AKT) signaling pathway (74). The mechanisms play an essential role in maintaining the structure of IVDs.

IVDD is associated with inflammation, immune cell infiltration, and neovascularization (2). These processes facilitate tissue repair in normal tissues. In the inflammatory microenvironment of IVDD, however, they exacerbate pain (75). Although the mechanisms by which T cells and macrophages are involved in the progression of IVDD are unclear, the passage of the disease may be due to the immune privilege of IVDs (62). The involvement of inflammatory factors and cytokines (such as IL-6, IL-1β, VEGF, and TNF-α) in the degeneration process can aggravate degeneration and cause pain (13). Therefore, reducing radicular pain and reversing the progression of IVDD by modulating the inflammatory microenvironment may be an effective treatment for IVDD.

## 3 Regulation of the inflammatory microenvironment

Protruding NP-secreted cytokines can worsen radicular pain and IVDD (33). Since there are no immune cell populations in the early stages of IVDD, the clearance of necrotic cells cannot be completed in a timely manner (13). Cells with phagocyte function are beneficial early in injury. Therefore, we hypothesize that infiltrating cells may perform specialized tasks in the healing processes of IVDs, whereas local phagocytosis-related cells fail to manifest at sufficient levels or at the right time (76). Therefore, the artificial intervention of cytokine release and induction of T cells and macrophages may be potential therapeutic strategies for IVDD.

### 3.1 Promotes the upregulation of anti-inflammatory factors

Transforming growth factor-β (TGF-β) is a multifunctional cytokine closely related to cell differentiation, apoptosis, and the maintenance of cell stability (77, 78). Many cells in the body, such as epithelial cells, tumor cells, immune cells, and stromal fibroblasts, can differentiate into inactive TGF-β complexes, which proteolytically leak TGF-β entities (79). There are three main isoforms of TGF-β (TGF-β1, -β2, and -β3) that cause different physiological responses after binding to their corresponding receptors (80). TGF-β receptors have many co-receptors in addition to the type I and II superfamily receptors (81). These receptors are widely present on the surfaces of body tissues and cells, making the body responses caused by TGF-β diverse (81). For example, TGF-β is a potent immunosuppressant and is closely related to the escape of cancer cells from immune system attacks (82). Studies have shown that TGF-β1 increases the contractility of myofibroblasts during wound healing, inhibits proteolytic enzymes in the ECM, and plays a crucial role in tissue remodeling (83). TGF-β1 further blocks IVDD by inhibiting the expression of chemokine (C–C motif) ligand 4 (CCL4) through extracellular signal-regulated kinase signaling (84). The inhibition of CCL4 by TGF-β1 occurs time- and dose-dependent. TGF-β1 can further inhibit the expression of IL-1β and TNF-α, thereby inhibiting the inflammatory response of IVDD (85, 86). The resolution of inflammation may therefore relieve the radicular pain caused by disc degeneration (87).

Systemic CCL5 is associated with discogenic back pain and moderate/severe IVDD. CCL5 can therefore be considered a biomarker for the diagnosis and monitoring of IVDD (88). Studies have confirmed that prominent NP can release CCL5 to induce MSCs to migrate into degenerated IVDs and promote their regeneration (89). Therefore, it is plausible that targeting CCL5 in IVDD could benefit disc repair. Zhou et al. used fibrin gel to target CCL5 around degenerated IVDs for their regeneration (38). CCL5 within fibrin gel is released around NF in a dose-dependent manner. Although CCL5 has a chemotaxis-homing effect on AF *in vitro* experiments, it does not promote IVDD regeneration in sheep IVDD models. This may have been because fibrin gel does not effectively encourage the movement of cell (38). In another study, Frapin et al. labeled pullulan with fluorescein isothiocyanate isomer I (FITC) (39). FITC-pullulan was stirred with NaOH solution and sodium trimetaphosphate to make pullulan microbeads (PMBs) carrying CCL5 to the degeneration sites. The PMBs adsorbed CCL5 and released 99% of it around the IVDs sustainably within 21 days. A significant increase in the migration distance of PMB-induced ADSCs to NPs and the number of ADSCs was observed on day 21 *in vitro* (39).

Since CCL5 cannot effectively induce TGF-β, the direct regulation of TGF-β may be beneficial to induce AF cells to differentiate and repair IVDs. Zhi et al. dissolved a prepared polyurethane (PU) fiber scaffold in an N, N-dimethylformamide solution and evaporated the solvent to form a PU film (40). The synthesis methods of the materials are shown in **Table 1**. Type I collagen fibers was used as cell carriers to encapsulate AF cells pretreated with TGF-β1 on PU membranes to create collagen-PU scaffolds. The advantage of PU scaffold is the continuous release of TGF and type I collagen to maintain the AF phenotype. *In vitro* experiments, the gene and protein expressions of AF cells were enhanced after induction by TGF-β1. It seems to indicate that AF cells pretreated with TGF-β1 may be a suitable cell source for the repair of AF ruptures (40). However, PU scaffolds have low porosity and are insufficient to carry a sufficient amount of TGF-β to maintain long-term effects. In addition, no animal experiments have proven this claim. Lerouge et al. mixed chitosan and porcine gelatin at a ratio of 3:2 to prepare chitosan hydrogels for loading NP cells (41). The peptide Link N (a peptide present in IVDs) outside the IVDs was further encapsulated in the hydrogels. The addition of Link polypeptide increased the NP encapsulation rate in hydrogels and increased NP deposition in a degenerative environment. Compared to the control group, the chitosan hydrogels induced more glycosaminoglycan (GAG) release from NP cells at 14 days *in vitro*. The increase in the number of GAGs was caused by the enhancement of NP cell activity. This increase in GAG release was nearly identical to that induced by TGF-β. This indicates that both induced AF and NP can repair IVDs. However, this is limited to early IVDD (90). In addition, most of these studies were conducted *in vitro*. It is, therefore, not yet known whether the effects *in vivo* are similar.

**Figure 2:**
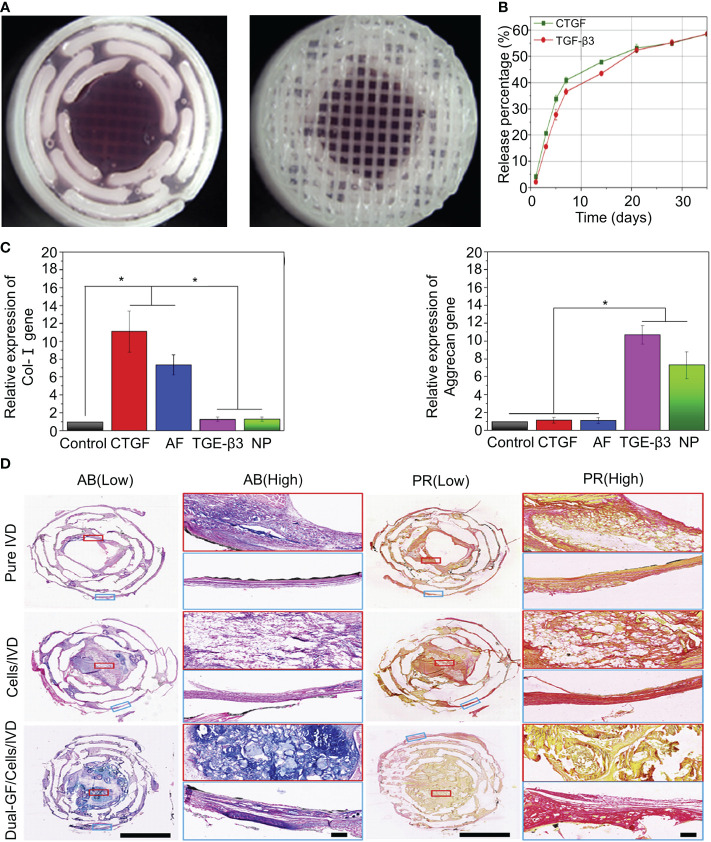
**(A)** Stereomicroscope images of the intermediate and surface structures of the 3D-printed scaffolds. **(B)** Release behavior of CTGF and TGF-β3 in the IVD scaffolds. **(C)** Expression of the type I collagen and aggrecan genes after inducing differentiation in different groups. **(D)** Histological staining of the intermediate layers of three IVD scaffolds with AB and PR dyes. Magnified images at low and high magnification. AB and PR dyes specifically stained GAG (blue) and type I collagen (red), respectively. Reproduced with permission from (42). *Indicates significant difference at a 99 %confidence level.

Connective tissue growth factor (CTGF) plays an essential role in extracellular matrix synthesis, especially in IVD (91). CTGF injections have been shown to promote the increased synthesis of human NP cells and induce decreased anti-inflammatory cytokines (92).Sun et al. fabricated PDA-NP scaffolds with dopamine and polycaprolactone (PCL) using 3D printing technology (**Figure 2**) (42). The synthesis methods of the materials are shown in **Table 1**. TGF-β3 and CTGF were loaded onto the surface of PDA-NP through covalent binding, forming a 3D-printed scaffold that could release dual growth factors for the reconstruction of NP and AF. CTGF is a cysteine-rich stromal cell protein involved in cell proliferation, differentiation, and connective tissue differentiation (93). PCL, which is widely used in 3D printing, was selected as the mechanical support for IVD scaffolds because of its good biodegradability and biocompatibility (94). PDA NPs have a diameter of 324.2 ± 13.9 nm. The loading efficiencies of CTGF and TGF-β3 for PDA NPs were 70.04 ± 0.94% and 72.34 ± 1.06%, respectively. PDA-NP released CTGF and TGF-β3 slowly and continuously (**Figure 2B**) and induced bone marrow stromal cells to differentiate into fibrocartilage-like cells (main components in the area around AF) and hyaline chondroid cells (main components in the area around NP). After 3 months of *in vivo* experiments, it was found that PDA-NP could effectively promote the expression of type I collagen, aggrecan (**Figure 2C**), and GAG in the AF and NP regions without causing apparent inflammation (**Figure 2D**) (42). However, not all fibrin types are good cellular carriers. For example, genipin-crosslinked fibrin (FibGen) is a viscous, high-modulus biomaterial that matches the properties of AF and exhibits low risk of hernias (95). FibGen is cytotoxic and induces the apoptosis of AF cells. In one study, the acute cytotoxicity of FibGen was reduced by modifying the binding sites of integrins to FibGen (43). The synthesis methods of the materials are shown in **Table 1**. The advantage of FibGen hydrogel is that the addition of integrins reverses apoptosis and increases TGF-β encapsulation (44, 96). The improved FibGen hydrogel carried TGF-β3 to repair AF *in vitro*. However, the toxicity of FibGen to normal AF still needs to be noted.

Although biomaterials loaded with TGF-β have achieved the ability to promote the reconstitution of NP and AF *in vivo*, this has only been verified in mice and not in large animals. Furthermore, TGF-β induces the differentiation of ADSCs into AF and NP cells only at the early stage of degeneration. As we have learned, IVDD is challenging to detect in the early stages, and most patients are diagnosed in the late stage when they have radicular pain. Therefore, whether TGF-β can reverse advanced IVDD remains unknown.

### 3.2 Inhibits the expression of inflammatory factors

#### 3.2.1 Interleukins inhibits

IL-1β is the most widely studied inflammatory factor in IVDD and is associated with inflammatory radiculopathy (13). IL-1β accelerates IVDD mainly by inducing the expression of MMPs in the ECM (35, 97). This process primarily depends on wingless-related integration site (Wnt)/β-catenin signaling (98). Studies have shown that the expression of Wnt1 and β-catenin in the NP of patients with lumbar disc herniation is significantly higher than that in regular patients. The activation of Wnt signaling induces senescence in NPC cells. Furthermore, the activation of Wnt/β-catenin signaling induces MMP expression, leading to increased ECM breakdown and IDD progression (99). Studies have confirmed that inhibiting Wnt/β-catenin signaling slows IVDD (100). As inflammatory factors can induce accelerated IVDD, the downregulation of local inflammation may restore IVDD.

**Figure 3:**
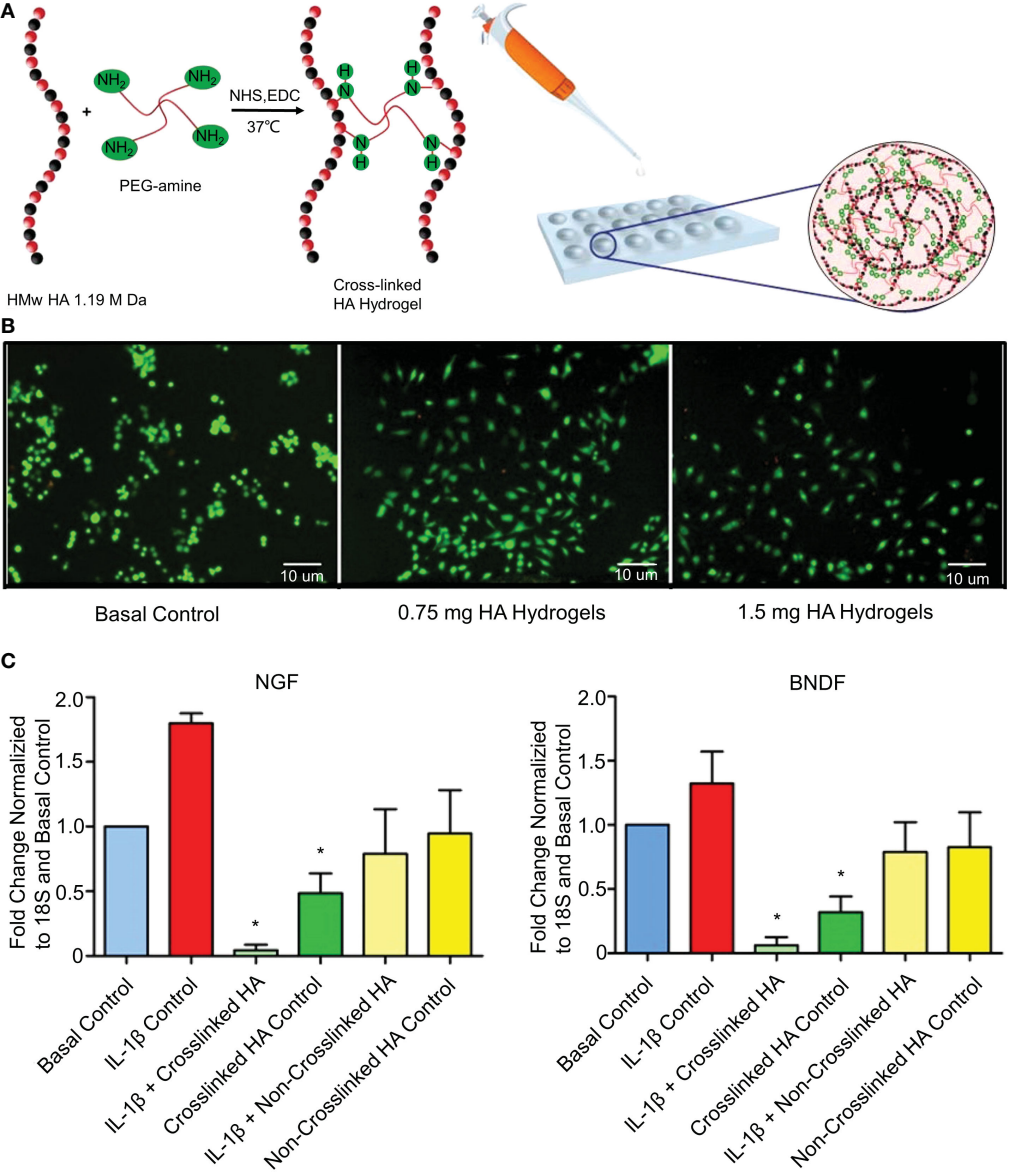
**(A)** Schematic diagram of the synthesis of the cross-linked HA hydrogel system. **(B)** Stained NP cell morphology by the LIVE/DEAD assay after 3 days of culture in the presence of hydrogels containing different doses of HA. Live cells are green (calcein staining) and dead cells are red (ethidium bromide staining). **(C)** Fold changes of NGF and BNDF downregulated in IL-1β-induced inflammation and normal NP cells treated with cross-linked HA hydrogels for 7 days. Reproduced with permission from (46). *Indicates significant difference at a 99 %confidence level.

Epigallocatechin 3-gallate (EGCG) exhibits anti-inflammatory, anti-catabolic, and antioxidant activities in IVD cells (101). EGCG interferes with the pro-inflammatory IL-1β cascade by reducing the activity of IRAK1–NF-κB/JNK/p38 signaling (101). However, oral EGCG does not increase intra-IVD EGCG concentrations, and high doses of EGCG increase hepatotoxicity and nephrotoxicity (102). Loepfe et al. used electrospray technology to encapsulate EGCG in porcine gelatin to produce gelatin microparticles (45). Electrospraying is a mild electrohydrodynamic encapsulation method that produces solid particles without needing high temperatures or toxic solvents. The average diameter of the gelatin microparticles was 661 ± 120 nm. The drug loading of gelatin microparticles reached 5.42 wt% and sustained the release of EGCG (80%) within 7 days. *In vitro* experiments confirmed that the gelatin microparticles significantly inhibited the expression of IL-1β. However, it has not been confirmed whether the gelatin microparticles have anti-inflammatory effects in the IVDD environment *in vivo*. A recent study further showed that IL-1β induces the mRNA expression of nerve growth factors (NGFs) and brain-derived neurotrophic factors (BDNFs) in degenerative NP cells (103). These neurotrophic factors have been shown to induce neural growth in IVDs (104). A normal IVD has no nerve tissue, and neuropathic pain occurs when neurotrophic factors induce nerve ingrowth into the IVD. Pandit et al. cross-linked high molecular weight (HMw)HA and 1-ethyl-3-(3-(dimethylamino)propyl)carbodiimide (EDC) to develop the cross-linked HA hydrogel system (**Figure 3**) (46). The synthesis methods of the materials are shown in **Table 1**. This study confirmed that the cross-linked HA hydrogel system with different doses of HA did not cause NP cell death *in vivo* (**Figure 3B**). The cross-linked HA hydrogel system could bind to the CD44 receptor on the surface of NPc and interfere with the binding of the CD44 receptor to IL-1β. After blocking the binding of IL-1β to CD44, the downregulation of NGF and BNGF mRNA expression provided a protective mechanism for inflammation-related pain (**Figure 3C**). However, the cross-linked HA hydrogel system only relieved neuropathic pain. It did not treat the underlying degeneration or restore the natural function of IVDs. This phenomenon may be caused by HA competing with IL-1β for its receptors. But this effect is weak enough to inhibit IL-1β completely. To increase the long-term inhibitory effect on IL1-β, Smith et al. mixed poly(lactic-co-glycolic acid) (PLGA) and IL-1 receptor antagonist (IL-1ra) to produce PLGA microparticles (47). The synthesis methods of the materials are shown in **Table 1**. *In vitro* experiments showed that PLGA microparticles could release IL-1ra continuously for 35 days. However, *in vivo* experiments demonstrated that PLGA microparticles effectively inhibited IL-1β for 7 days and that the effect of IL-1ra beyond 7 days was to reduce GAG loss. This difference is not due to insufficient IL-1ra but to a lower pH at the release site due to PLGA degradation. Therefore, the degradation properties of biomaterials are essential, and we must develop biomaterials that are more suitable for substance delivery.

Chitosan is a natural, biocompatibile, and biodegradable polysaccharide that is mainly used in drug delivery systems and tissue engineering (105). The chitosan surface polarizes macrophages into the M2 phenotype without causing significant T cell proliferation. Goncalves et al. prepared nanoparticles using a combination of chitosan and poly γ-glutamic acid (γ-PGA) for the coacervation method (48). Diclofenac (Df) was added to the nanoparticles to form Ch/Df/γ-PGA NP. The diameter of the nanoparticles was 175 ± 32 nm. The Df reduces the local inflammatory response and the production of pro-inflammatory cytokines (IL-1β and IL-6) by inhibiting the cyclooxygenase (COX)-2 pathway. In addition, down-regulation of MMPs expression in ECM was detected 7 days after NP injection, and IVDD was remodeled *in vitro*. The Ch/Df/γ-PGA NP has promising prospects for development. However, animal experiments were not performed in this study, and it is not yet known whether Ch/Df/γ-PGA NP was advantageous in IVD anti-inflammatory activity and the remodeling of IVDD.

**Figure 4:**
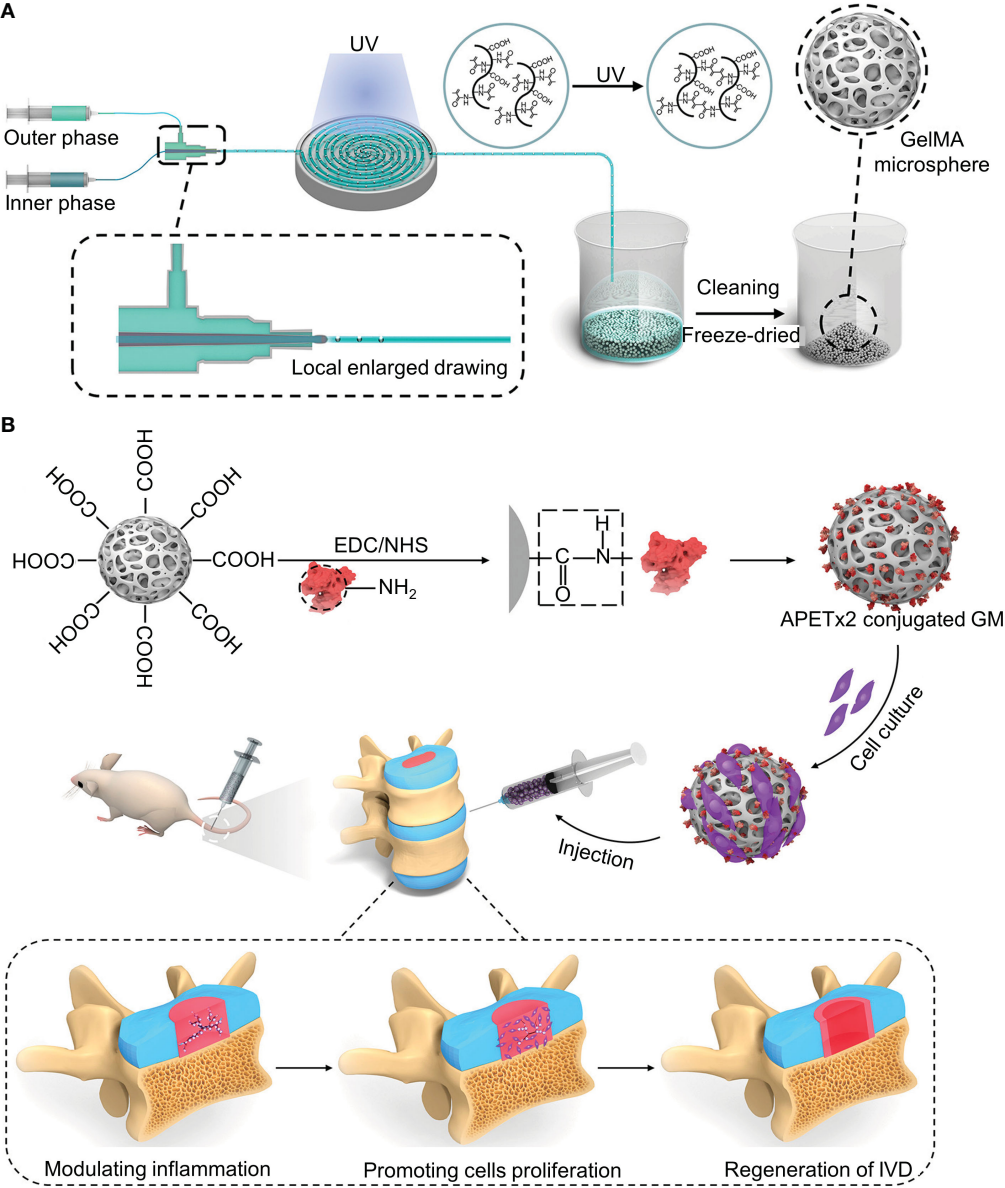
**(A)** Schematic diagram of the synthesis of the GelMA microsphere. **(B)** The carboxyl on the microsphere surface was activated by EDC/NHS, followed by being covalently linked to the amino residue on the APETx2 to form APETx2-GelMA microsphere (GA). Reproduced with permission from (108).

Acid-sensing ion channels (ASICs) are expressed in the nervous system and are mainly activated by extracellular acid regulation (106). ASIC3 is mainly expressed in the peripheral nervous system and, to a lesser extent, in the central nervous system (CNS) (107). Hyperactivation of ASIC-3 induces the expression of proinflammatory cytokines in the NPs. APETx2 is an antagonist of ASIC3. Blocking ASIC3 reduces local inflammation and reduces inflammatory pain (107). Bian et al. designed a “peptide-cell-hydrogel” GelMA microsphere for the control of intervertebral disc inflammation (108) (**Figure 4A**). The carboxyl on the microsphere surface was activated by EDC/NHS, followed by being covalently linked to the amino residue on the APETx2 to form APETx2-GelMA microsphere (GA) (**Figure 4B**). NP cells were premixed with GA to make a “peptide-cell-hydrogel” GelMA microsphere (GNA). The hydrogel has an elastic modulus of 25.23 ± 2.58 kPa, which can protect NP cells from shear damage. Detected at week 8 after GNA infusion into degenerate discs in mice, GNA downregulated ASIC-3 expression, decreased peripheral IL-1β expression, increased proliferative activity, and enhanced ECM deposition

IL-17 is a crucial characteristic cytokine of T-helper 17 (Th17) cells. Th17 is the primary source of IL-17. The innate immune system can further secrete IL-17 (109). IL-17 is associated with inflammation, and increasing evidence has shown that IL-17 is involved in the occurrence and progression of IVDD (110–112). There is no IL-17 in normal AF and NP, and elevations of IL-17 can be detected in degenerative structures (113). Importantly, the expression of IL-17 in IVD tissues has been found to increase with the severity of IVDD. Therefore, IL-17 levels may be associated with the extent of IVDD (112). IVDD exposure to TNF-α stimulates the production of IL-17, which promotes the degradation of the ECM mainly through the IL-17A receptor-nuclear factor kappa B (NF-κB) pathway (114). IL-17A receptors are mainly distributed on the surfaces of NP cells; therefore, the inhibition of IL-17A receptors can attenuate ECM degradation and delay IVDD. This approach has not been reported so far, but we speculate it may be a new target for slowing IVDD.

Biomaterials for the treatment of IL-regulated IVDD have also been reported. However, the studies were primarily performed *in vitro* with cellular experiments to verify its role in IVDD. There are a few reports of 3D-printed biomaterials that simulate IVDD in mice, and this scaffold has been confirmed to have good support and regulation. However, this phenomenon has not been previously reported in large animals. The developed nanoparticles are anti-inflammatory to IVDD and promote ECM regeneration *in vitro*. However, most of these nanoparticles require local injections. The high pressure inside NP makes NP cells unable to withstand the pressure of the nanoparticle injection. Therefore, our future research direction is not only toward finding new targets but also toward developing new biomaterials to repair IVDD.

#### 3.2.2 Tumor necrosis factors

Similar to IL-1β, TNF-α is a pro-inflammatory cytokine that belongs to the TNF ligand superfamily (115). TNF-α can trigger inflammatory responses *in vivo* and induce the expression of IL-1β, IL-6, IL-8, and IL-17 (116). IL-17 has been linked to the severity of IVDD (112). Prominent NP can release CCL5 to induce MSC migration into IVDD to promote IVD regeneration (89). However, studies have shown that TNF-α can recruit more inflammatory factors by inducing CCL5 differentiation (117). In addition to promoting inflammatory cells, TNF-α can increase MMP expression through the NF-κB/MAPK signaling pathway to degrade the ECM (118). Recently, it has been shown that the secretion of TNF-α by degenerated IVDs induces senescence in NP cells that induce senescence in healthy cells by inhibiting Stat3 phosphorylation through paracrine effects (119). Similar to IL-1β, TNF-α can accelerate IVDD. Therefore, the inhibition of TNF-α may delay neuropathic pain.

**Figure 5:**
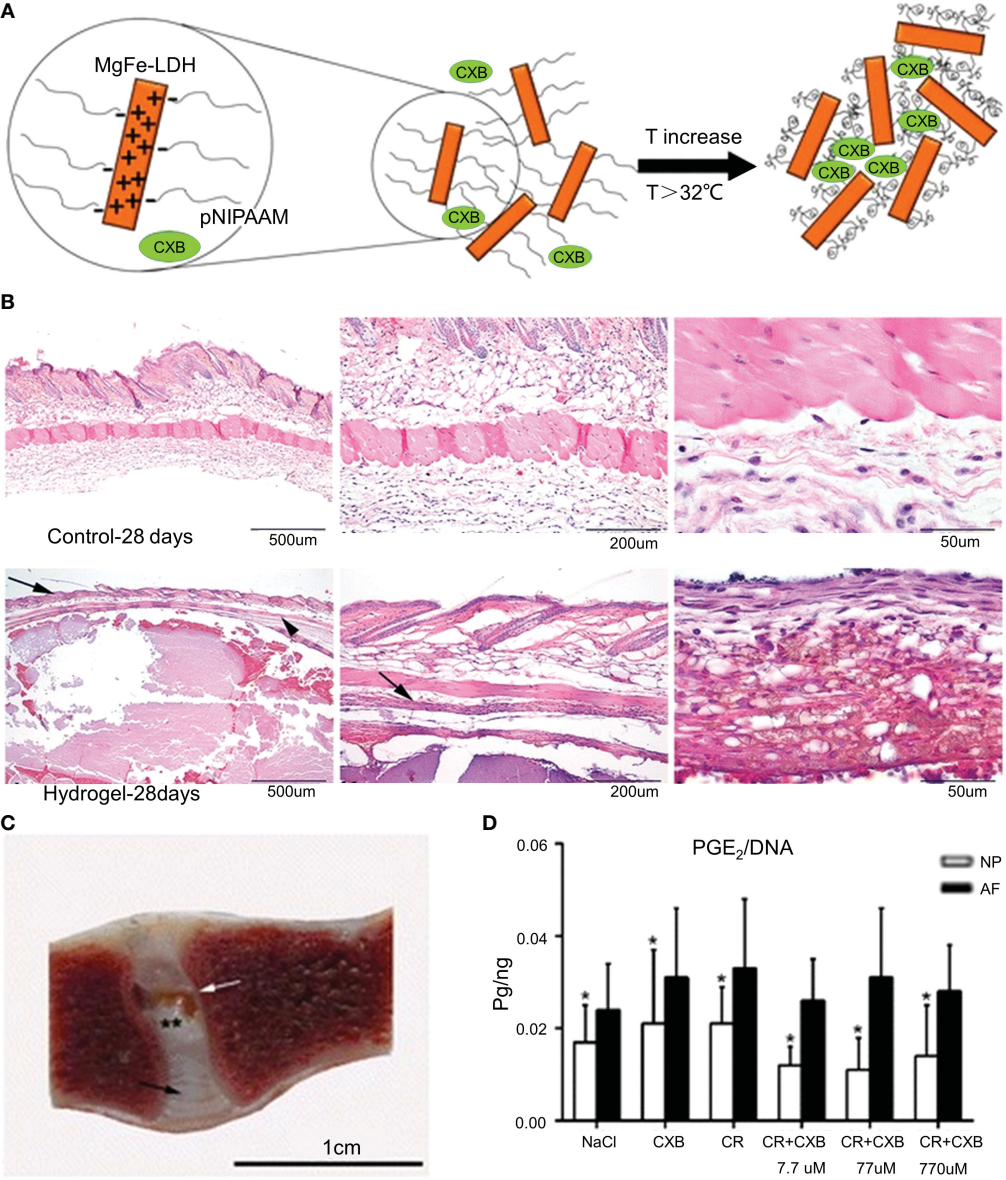
**(A)** Schematic diagram of the synthesis of the pNIPAAM hydrogel. **(B)** Histological staining (hematoxylin and eosin staining) of the hydrogel and control group after 28 days in mice. The epidermis and flesh lipid membranes are indicated by the arrows. **(C)** One month after the intradiscal injections, the pNIPAAM hydrogel (white arrow) was visible in the NP. **(D)** The DNA level in PGE 2NP was significantly lower than that in AF in all treatments. Reproduced with permission from (50). *Indicates significant difference at a 99 % confidence level P ≤ 0.05. **Indicates significant difference at a 99 % confidence level P ≤ 0.01.

Curcumin is derived from the rhizomes of natural herbs and has anticancer, antibacterial, and anti-inflammatory properties (120). Studies have shown that curcumin inhibits TNF-induced NF-κB activation and downregulates the release of pro-inflammatory factors (121). However, the low solubility of curcumin and its poor absorption into systemic circulation limit its clinical applications (122). Setton et al. modified curcumin with carbamate and coupled it with an elastin-like polypeptide (ELP) to obtain a biodegradable ELP-curcumin conjugate (49). The synthesis methods of the materials are shown in **Table 1**. ELP-curcumin conjugates have a high drug load, rapidly releasing curcumin *in vitro* and maintaining its biological activity. In an *in vitro* study, curcumin was continuously removed from the conjugate. After 96 h, 55% of the loaded curcumin had been released. The curcumin content around the nerve was detected at 48 h, and the amount of curcumin released in the conjugate was five times that of free curcumin. This suggests that the conjugate could reduce the adverse factors of the poor systemic absorption of curcumin and effectively deliver curcumin to the periphery of nerves to alleviate peripheral inflammatory responses. Further studies have shown that TNF-α can upregulate the expression of prostaglandin E 2 (PGE2) in NP cells. PGE2 inhibits proteoglycan synthesis, thereby degrading the ECM (123). Willems et al. loaded celecoxib (CXB) into a poly-N-isopropylacrylamide (pNIPAAM) polymer (**Figure 5**) (50). The synthesis methods of the materials are shown in **Table 1**. CXB is a COX-2 inhibitor that can specifically block the production of PGE2. CXB-containing hydrogels effectively released CXB and continuously inhibited PGE2 (52%) *in vitro*. Twenty-eight days after the mice were injected with the hydrogel, the pNIPAAM polymer formed a denser fibrous structure than the blank control group (**Figure 5B**). Reductions in spinal reflexes were detected on the day after the infusion of the CXB-filled hydrogels into dogs. The hydrogel with CXB spontaneously formed a solid at 37°C without leakage after the injection (**Figure 5C**). The pNIPAAM polymer significantly reduced PGE2 in NP compared to AP (**Figure 5D**). This was consistent with the results of previous studies (123). Interestingly, this was one of the few *in vivo* experiments conducted in large animals to demonstrate the efficacy of hydrogels. However, similar to IL, the reduction of inflammatory responses by inhibiting TNF-α to modulate the microenvironment remains limited to early IVDD. There have been no reports relating to late IVDD.

### 3.3 Oxidative stress correlated factors

#### 3.3.1 Hypoxia-inducible factors

HIF is a transcription factor expressed in cells in response to hypoxia. HIF-1 consists of a constitutively expressed subunit, HIF-1β, and another subunit, HIF-1α, which is regulated by cellular O concentration (124). HIFs have strong anti-apoptotic effects and are involved in regulating cellular responses to oxidative stress (125). NP cells have been reported to express HIF-1α to maintain cellular energy and ECM metabolism independent of oxygen tension (126). Endoplasmic reticulum oxidative stress in IVDD can accelerate IVDD progression, and HIF can reduce endoplasmic reticulum oxidative stress in IVDD (127). Studies have shown that the knockout of the HIF-1α gene in mouse NP cells results in massive death and the loss of HIF-1α results in reduced collagen II and proteoglycan protein expression in IVDs (128, 129). Therefore, HIF-1α is expressed by NP cells and has a protective effect on IVDs.

Cells with degenerated NP or AF have multi-differentiation potential and can be stimulated to repair IVDs (130). However, NP cell-derived disc progenitor cells (DPCs) are sensitive to hypoxia and fail to upregulate HIF to undergo apoptosis under hypoxia (125). Huang et al. used small leucine-rich proteoglycans (SLRPs) and biglycan to provide growth substrates for DPCs (51). SLRPs can bind and regulate TGF-β to activate the MAPK pathway, and TGF-β can enhance HIF-1α expression through TGF-β receptor (ALK5) kinase activity. It has been further confirmed that IVDD can be repaired by adjusting HIF *in vitro*. However, there are currently no studies showing the effects on animals. Chitosan itself can reduce the apoptosis of NP owing to oxidative stress (131, 132). Studies have shown that the enhancement of the PI3K/Akt pathway by chitosan protects cells against apoptosis (131). Zhang et al. mixed chitosan and alginate at a ratio of 1:1. The mixture was phacoemulsified to obtain a C/A gel scaffold (52). Alginate is also a safe biodegradable polysaccharide material with biocompatibility, solubility, porosity, and tunability of viscosity and concentration (133). The C/A gel scaffold has a porosity of 80.57%, which can effectively load ADSCs and provide a good framework for their growth. Morphological changes can occur during gel scaffold degradation, such as shrinkage, increased transparency, and 3D morphology. The advantage of the C/A gel scaffold is that it can provide ADSCs with a hypoxic environment similar to NP cells during *in vitro* degradations. This hypoxic environment favors the differentiation of ADSCs into NPs. After 21 days of culture *in vitro*, the C/A gel scaffold induced a significantly higher mRNA expression of HIF-1α than the normoxia-induced group. It increased the number of type II collagen fibers in the ECM. C/A gel scaffolds can generate the same functional ECM as standard NP, providing a basis for the active recovery of IVDD.

Calcification is frequently associated with IVDD, a condition of osteophyte growth at the peripheries of endplates, and vertebral margins (125). This is mainly due to the deposition of calcium-containing substances in the ECM outside the IVD. Studies have shown that HIF-1α can inhibit the secretion of inorganic pyrophosphate by NP and reduce the calcification of IVDs (134). Calcification can aggravate IVDD. miR-199a is an endogenous small non-coding RNA that directly targets HIF-1α and downregulates HIF-1α expression (135). Thus, anti-miR-199a delivery specifically inhibited endogenous miR-199a and enhanced Hif-1α expression to inhibit the calcification of IVDs. Feng Ganjun et al. developed poly (L-lactic acid) graft poly(hydroxyethyl methacrylate) (PLLA-g-PHEMA) nanoparticles using a phase separation method (53). The synthesis methods of the materials are shown in **Table 1**. The diameter of the nanoparticles was between 30 and 60 μm. Nanoparticles as non-viral vectors for miRNA delivery can efficiently deliver miRNAs into cells and bone marrow MSCs (BMMSCs) together in the ECM. BMMSCs promote the regeneration of aging NP. The detection of nanoparticles at week 12 in mice reduced calcification of ECM and enhanced HIF-1α-induced BMMSCs to promote mouse NP cell regeneration.

#### 3.3.2 Reactive oxygen species

**Figure 6:**
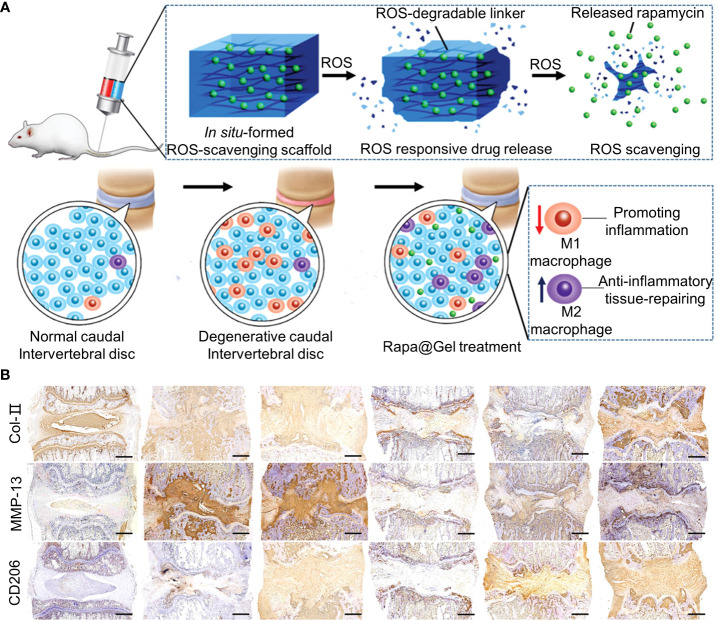
**(A)** Schematic representation of Rapa-loaded ROS-responsive hydrogels. **(B)** Immunohistochemical staining for collagen type II (COL2), matrix metalloproteinase 13 (MMP13), and CD206 at week 12. Immunohistochemical staining showed that COL2 expression was upregulated, MMP13 expression was downregulated, and CD206 expression was upregulated in the rapamycin-treated group compared with the degenerative control group. Reproduced with permission from (55).

In addition to inflammatory cells, oxidative stress caused by high levels of ROS is a hallmark of chronic inflammation (136). Oxidative stress damages vital cellular structures, leading to apoptosis and senescence (54). The exposure of NP to ROS releases IL-1β, triggering a cascade of reactions that exacerbate IVD damage (137). High levels of ROS also inhibited HIF-α expression. ROS is regulated by glutathione, superoxide dismutase, and catalase (138). Therefore, blocking ROS production may be beneficial for the repair of IVDD. An ROS-labile linker was synthesized *via* the quaternization reaction of N1,N1,N3,N3-tetramethylpropane-1,3-diamine with an excess of 4-(bromomethyl) phenylboronic acid (**Figure 6**) (55). The synthesis methods of the materials are shown in **Table 1**. The ROS-labile linker was cross-linked with vinyl alcohol (PVA) to form a ROS-scavenging hydrogel for loading rapamycin. In a ROS-rich environment, the ROS-scavenging hydrogel was degraded by the consumption of ROS to release rapamycin. Rapamycin has immunosuppressive effects and induces apoptosis. Rapamycin hydrogel clears ROS and promotes macrophage polarization to M2, reducing inflammatory responses *in vitro*. At week 12 of the *in vivo* IVDD model, rapamycin hydrogels downregulated MMP-13 in the ECM and upregulated type II collagen (**Figure 6B**).

Pandit et al. prepared calcium carbonate, poly (allylamine hydrochloride) (PAH), and tannic acid (TA) into polymer capsules using a layer-by-layer approach (54). TA is a natural polyphenol that has shown a considerable capacity to scavenge a wide variety of free radicals and pro-oxidant molecules owing to its redox properties (139). Calcium carbonate was used as the core of the polymer capsule to load catalase. TA accelerated the release of catalase while scavenging ROS on the surface. The TA-functionalized polymer capsules promoted the release of hydrogen peroxide more effectively than the non-TA-functionalized capsules. The study confirmed that the encapsulation rate of hydrogen peroxide in the polymer capsule was 97.8%, which was five times higher than that of physical adsorption. Compared with 67% of oxidative stress-positive NP cells in the blank group, this double ROS-scavenging polymer capsule reduced oxidative stress-positive NP cells to 3% *in vitro*. In addition, polymer capsules can downregulate the expression of MMPs in the ECM. Although the polymer capsules exhibited the ability to induce the regeneration of IVDs *in vitro*, it has not been verified whether a similar effect occurs *in vivo*.

IVDs are non-neurovascular structures, and the growth of NP cells is adapted to the hypoxic state. However, studies have demonstrated that hypoxia in NP cells may exacerbate IVDD (140, 141). A particular concentration of oxygen appears to be beneficial to NP cells. Perfluorotributylamine (PFTBA) belongs to the perfluorocarbon family with high oxygen solubility and can modulate the oxygen tension of IVDs (142). Zhen et al. used PFTBA to prepare scaffolds to load alginate. Alginate, a widely used scaffold for the regeneration of IVDs, has shown better ECM deposition than other materials (56). The synthesis methods of the materials are shown in **Table 1**. The PFTBA concentration in the scaffolds was proportional to the oxygen concentration in the ECM. This study confirmed that the addition of PFTBA (2.5–10%) under hypoxic conditions significantly reduced the rate of cell death in NP cells *in vitro*. When simulating IVDD in mice, it was found that 2.5% of the PFTBA scaffolds induced more type II collagen and proteoglycans in the ECM at 6 weeks compared with other concentrations of the PFTBA scaffolds. Another advantage of the PFTBA-alginate scaffolds was that their stability did not change as PFTBA degraded. However, the study did not measure the oxygen levels *in vivo*. Furthermore, IVDD in mice results from spontaneous terminal calcification, the mechanism of which remains unclear. Therefore, it is necessary to investigate further whether regulating the oxygen content of NP cells in animals is beneficial for the repair of IVDD.

## 4 Discussion

Normal IVDs are immune-privileged tissue structures. Degeneration occurs when IVDs are stimulated by inflammation, oxidative stress, and stress. IVDD recruits T cells and macrophages to clear necrotic tissues and cellular debris. Furthermore, the average NP and AF cells of IVDD become targets of immune system attacks, thereby aggravating IVDD. We found that repairing IVDD by modulating the inflammatory microenvironment is a viable therapeutic modality. However, owing to the lack of vascular structures in IVDs, the biological factors that regulate immunity cannot effectively reach the sites of IVDD. In addition, the lack of blood vessels in IVDs means that natural characteristics cannot obtain the nutrients they need to repair degenerated IVDs. Therefore, spontaneous repair of IVDD seems impossible. In recent years, biomaterials that regulate the inflammatory microenvironment to improve IVDD have been reported. For example, chitosan, curcumin, and alginate can protect NP cells and the ECM and act in gels to carry biological regulators to repair IVDD. Engineered nano scaffolds can also delay IVDD while preserving the survival of bioregulators by delivering them around IVDs. These biomaterials have shown advantages in inducing stem cell differentiation and reducing the inflammatory cascade induced by IL-1β and TNF-α. Still, they have also downregulated the inflammatory response by scavenging ROS in the ECM. The biomaterials can also reduce MMP expression and provide a favorable environment for IVD repair. These advantages indicate that regulating the inflammatory microenvironment by biomaterials to repair IVDD may be helpful in clinical treatments.

Although it seems possible that biomaterials regulate inflammation-immune environment repair IVDD, there are two significant problems worth noting. First, most of the studies on IVDD were performed under conditions that mimicked IVDD *in vitro*. In line with the studies conducted on mice and dogs, biomaterials can adapt to *in vivo* stress and histocompatibility and play a positive role in the repair of IVDs. However, there are few studies on regulating IVDD repair in the inflammatory microenvironment *in vivo*. Further validation is needed to confirm the strategies involving biomaterials to modulate the inflammatory microenvironment *in vivo*, especially in large animals. Second, the use of biomaterials in regulating the inflammatory microenvironment to repair IVDD is only significant in the early stage of the disease. However, most cases of IVDD are challenging to detect early on when the patients are asymptomatic. However, it is unknown whether biomaterials modulate the inflammatory microenvironment to repair IVDD in patients with advanced IVDD.

## Author contributions

All authors read and approved the final manuscript. YX wrote the initial manuscript. CF and YX contributed new ideas. HW created the figures. YL and RY created **Table 1**. YH and JZ made the english language editing. YX, YL, and RY revised the manuscript and approved the final version.
